# The vascular phenotype in Pseudoxanthoma elasticum and related disorders: contribution of a genetic disease to the understanding of vascular calcification

**DOI:** 10.3389/fgene.2013.00004

**Published:** 2013-02-12

**Authors:** Georges Lefthériotis, Loukman Omarjee, Olivier Le Saux, Daniel Henrion, Pierre Abraham, Fabrice Prunier, Serge Willoteaux, Ludovic Martin

**Affiliations:** ^1^PXE Health and Research Centre, University Hospital of AngersAngers, France; ^2^L’UNAM, UMR CNRS 6214 - Inserm 1083, Medical School of AngersAngers, France; ^3^John A. Burns School of Medicine, University of HawaiiHonolulu, HI, USA; ^4^L’UNAM, EA3860, Medical School of AngersAngers, France

**Keywords:** pseudoxanthoma elasticum, calcium, vessels, cardiovascular diseases, elasticity, ankle-brachial index

## Abstract

Vascular calcification is a complex and dynamic process occurring in various physiological conditions such as aging and exercise or in acquired metabolic disorders like diabetes or chronic renal insufficiency. Arterial calcifications are also observed in several genetic diseases revealing the important role of unbalanced or defective anti- or pro-calcifying factors. Pseudoxanthoma elasticum (PXE) is an inherited disease (OMIM 264800) characterized by elastic fiber fragmentation and calcification in various soft conjunctive tissues including the skin, eyes, and arterial media. The PXE disease results from mutations in the ABCC6 gene, encoding an ATP-binding cassette transporter primarily expressed in the liver, kidneys suggesting that it is a prototypic metabolic soft-tissue calcifying disease of genetic origin. The clinical expression of the PXE arterial disease is characterized by an increased risk for coronary (myocardial infarction), cerebral (aneurysm and stroke), and lower limb peripheral artery disease. However, the structural and functional changes in the arterial wall induced by PXE are still unexplained. The use of a recombinant mouse model inactivated for the Abcc6 gene is an important tool for the understanding of the PXE pathophysiology although the vascular impact in this model remains limited to date. Overlapping of the PXE phenotype with other inherited calcifying diseases could bring important informations to our comprehension of the PXE disease.

## ARTERIAL CALCIFICATION IS AN INDEPENDENT RISK FACTOR OF CARDIOVASCULAR DISEASES

Arterial calcification is gaining an increasing interest as an independent marker for cardiovascular (CV) diseases in acquired metabolic diseases, such as type II diabetes (Becker et al., 2008) and chronic renal insufficiency (Goodman et al., 2000). Vascular calcification increases physiologically with age and studies from Egyptian mummies have revealed that arterial calcification is not a feature of modern life style due to the absence of risk factors such as smoking, high fat cholesterol diets encountered in these ancient civilizations (Allam et al., 2011).

Calcification of the intimal layers can complicate atherosclerotic plaques favoring the risk of rupture whereas deposit within the medial layer contributes to stiffen arterial wall leading to hypertension, cardiac hypertrophy, and heart failure (Demer and Tintut, 2008). Similarly to the bone, ectopic calcium sediment within the arterial wall is a dynamic and tightly regulated biological process involving a large number of cytokines and cellular pathways (Giachelli, 2004; Persy and D’Haese, 2009). Contrary to the bones, the artery is a tubular organ that should remain soft and flexible but resilient to the high distending blood pressure. The elastic properties of the arterial wall plays a key role in damping the cyclic pressure changes produced by the beating heart (O’Rourke et al., 2002). This dampening effect predominates within the large elastic vessels such as aorta and progressively decreases downstream as the vessel wall becomes more muscular in the medium and small-sized arteries allowing a continuous flow and to protect the thin walled capillaries against high pressures. Therefore, any changes in arterial wall elasticity, i.e., the recoiling force, and distensibility, (the capacity to be distended), either physiologically with aging (so called arteriosclerosis) or in response to abnormal metabolic conditions such as type II diabetes or chronic renal insufficiency, contribute to stiffen the arterial wall. Stiffening of the arterial wall will reduce the dampening effect leading to the increase in systemic arterial pressure, mainly pulse pressure, and ultimately damage the small capillaries in end-organs such as brain, kidneys, or heart (Laurent et al., 2009).

Our understanding of the direct or indirect contribution of calcification in the vascular system remains limited due to the multifactorial mechanisms. Although both elastic lamina and medial calcification could share similar genetic determinants, whether or not calcification precedes or follows the disruption and/or the degeneration of the elastic fibers is often difficult to establish (Wang et al., 2009). The resulting elasto-calcinosis refers to a timely and well-balanced interaction between several local and remote factors (see review Atkinson, 2008). The mechanisms underlying calcification of the elastic fibers are multifactorial including physico-chemical conditions, inflammation and oxydative stress, metabolic dysfunction, and unbalanced promoters/inhibitors of calcification. This process can occur focally in the intimal layer and may complicate atheromatous plaques whereas it occurs more diffusely in the media.

The role of genetics in the calcification process is likely to take an important place since than >40% of the variance of aortic and coronary calcification phenotype could be under the control of genes. The roles of genes have been deciphered in various monogenic diseases but also in the general population (O’Donnell et al., 2002; Assimes et al., 2008; Rampersaud et al., 2008) and have been recently reviewed by Rutsch et al. (2011).

The pathophysiology of calcification in metabolic diseases is of a particular concern and the present review will focus mainly on pseudoxanthoma elasticum (PXE), an inherited disease displaying specific and unusual characteristics that belongs to a larger group of genetically and metabolically determined calcifying vascular diseases.

## PSEUDOXANTHOMA ELASTICUM: AN ENIGMATIC CALCIFYING GENETIC DISEASE

Pseudoxanthoma elasticum is an inherited autosomal recessive multisystem disorder affecting connective tissues. Its phenotypic expression is characterized by the fragmentation and mineralization of elastic fibers in the skin termed elastorrhexis, the Bruch’s membrane of the retina and the vasculature (Neldner, 1988; Hu et al., 2003; Uitto et al., 2011). Its prevalence is estimated from 1/25,000 to 1/50,000 and the causative mutations have been identified in the *ABCC6 *gene encoding a trans-membrane ATP-binding cassette transporter, subfamily C, member 6 (ABCC6/MRP6; Le Saux et al., 2000) that is primarily expressed in the liver and the kidney, but with much lower expression in other affected tissues such as skin, eyes, or arteries (Kool et al., 1999). The biological function of the ABCC6 transporter and its substrates remains totally unknown to date. The phenotype seems to result from an unknown defect originating from the liver and the kidney leading to the extracellular calcium sediment but probably intracellular (Martin et al., 2012). Several studies have demonstrated that normal tissues exposed to the serum from PXE patients or mice knockout for Abcc6 are able to calcify (Le Saux et al., 2006; Jiang et al., 2007, 2010). Therefore, PXE is considered as a prototypical metabolic calcifying disease of genetic origin (Jiang and Uitto, 2006). PXE is also characterized by its delayed onset and a variability in its phenotypic expression suggesting that a large number of co-factors contribute to its phenotype. The classical risk factors involved in arteriosclerosis, such as tobacco, hypertension, dyslipidemia, could be greatly suspected to interfere with the severity of the vascular expression of the disease, although most of the vascular complications in PXE occur later during the life (>40 years) than the skin and eyes lesions, with an unexplained female preponderance (Uitto et al., 2010). The 2/3 female–1/3 male ratio in PXE leads to an unusual and unexplained prevalence of arterial disease in female compared to the general population. A PXE-like phenotype has also been reported in other genetic diseases such as beta-thalassemia and sickle cell anemia (Fabbri et al., 2009), cutis laxa (Vanakker et al., 2007), generalized arterial calcification in infancy (Kalal et al., 2012), a defect in gamma-glutamyl carboxylase (Vanakker et al., 2007), familial idiopathic basal ganglia (Wang et al., 2012) and more rarely induced by pharmacological substances such as seen with D penicillamine (Ratnavel and Norris, 1994). The fact that PXE phenotype could overlap with other genetic diseases suggests that these diseases share a common pathophysiology (Nitschke et al., 2012). The main phenotypical differences reported in the literature between the genetic calcifying diseases are summarized in **Table 1**.

**Table 1:** Comparative characteristics of the arterial phenotype in PXE and other related disorders.

	Beta-thalassemia/Sickle cell disease	PXE-like + cutis laxa	PXE	GACI	ACDC (or CALJA)
**Elementary arterial phenotype**
Intima-media thickness	Increased	Unknown	Increased		
Endothelial dysfunction	Defective	Unknown	Unknown	Unknown	Unknown
Stenosis	Yes (26.7% mostly cerebral)	Yes (PAD 50%)	Yes (mostly coronary and cerebral)	Yes (mostly coronary, cerebral, and renal)	
Dilatation/aneurysm	Sporadic	Yes (mostly cerebral)	Infrequent		Popliteal
Calcification	Present	Present	Present	Present	Present
Arterial malformations			gastrointestinal, eyes (neovascularization) and carotids (?)		
Coagulation defects	Present (50%)	Present	Secondary?	Present	Present
Defective gene	HBB/HBF	GGCX	ABCC6	ENPP1	NT5E

*For each disorder, bibliographical references for vascular phenotype are indicated. GACI, generalized arterial calcification of infancy (Rutsch et al., 2001; Kalal et al., 2012); ACDC, arterial calcification or CALJA, calcification of joints and arteries due to deficiency of CD73 (Markello et al., 2011); HBB/HBF, hemoglobinopathies Beta and S (Aessopos et al., 1998; Fabbri et al., 2009; Musallam et al., 2011); GGCX, gamma-glutamyl carboxylase ( Vanakker et al., 2007, 2011; Li et al., 2009a); ABCC6, adenosine triphosphate-binding cassette (ABC) gene subfamily C (Boutouyrie et al., 2001; Germain et al., 2003; Kornet et al., 2004; Leftheriotis et al., 2011; Kupetsky-Rincon et al., 2012); ENPP1, ectonucleotide pyrophosphatase/phosphodiesterase (Mackenzie et al., 2012; Nitschke et al., 2012); NT5E, Ecto-5′-nucleotidase (Markello et al., 2011; St. Hilaire et al., 2011).*

A number of candidate substrates for ABCC6 have been hypothesized. The observation that PXE patients exhibit a low plasma vitamin K level and that anti-vitamin K drugs accelerate calcification in normal and in Abcc6^-^^/^^-^ mice (Li et al., 2012) raised the hypothesis for a role of vitamin K in the calcification process. Vitamin K is a key factor for the activation of tissue calcification inhibitor factors such as matrix Gla proteins (MGPs). This hypothesis has not been confirmed due to no changes in the calcification process occurring in PXE animal models supplemented with vitamin K (Brampton et al., 2011; Fülöp et al., 2011; Gorgels et al., 2011). The implication of adenosine as a calcifying factor in PXE has also been hypothesized (Markello et al., 2011), as well as oxidative stress (Pasquali-Ronchetti et al., 2006; Zarbock et al., 2007), although the role of oxidative stress has not been evidenced by endothelial dysfunction at present in PXE.

## THE VASCULAR LESIONS AND HISTOLOGICAL FINDINGS IN PXE

The elementary arterial lesions observed in PXE are characterized by mineralization of the elastic fibers of the medial layer, predominantly within the medium and small-sized musculo-elastic arteries. Abnormal elastic fibers are thought to be produced by the PXE skin fibroblasts (Quaglino et al., 2000), but could also occur with normal fibroblasts in the presence of PXE serum (Le Saux et al., 2006) or in presence of elastin degradation products (Simionescu et al., 2005). These findings suggest that PXE is a disorder of the mechanisms controlling the production of matrix constituents and that elastic fiber mineralization is caused by factors abnormally produced and entrapped within the fiber during elastin fibrogenesis (Baccarani-Contri et al., 1994). These finding called for the “elastosis hypothesis” as the primary mechanism in PXE and that calcification is secondary. A primary role for smooth muscle cells and fibroblasts in PXE is suspected as they are a source of many regulatory proteins involved in the calcification process such as alkaline phosphatase and MGP (Shanahan et al., 1999; Simionescu et al., 2005). Finally, an abnormal balance between elevated proteolysis activity with increased P-selectin (Gotting et al., 2008), matrix metallo-proteinase (MMP) 2 and 9 (Diekmann et al., 2009), suggests an abnormal remodeling of the extra cellular matrix (ECM) in PXE. Despite unknown mechanism sequence for elastosis and calcification, the nature and affected sites of calcification lead to different functional expression.

**FIGURE 1:**
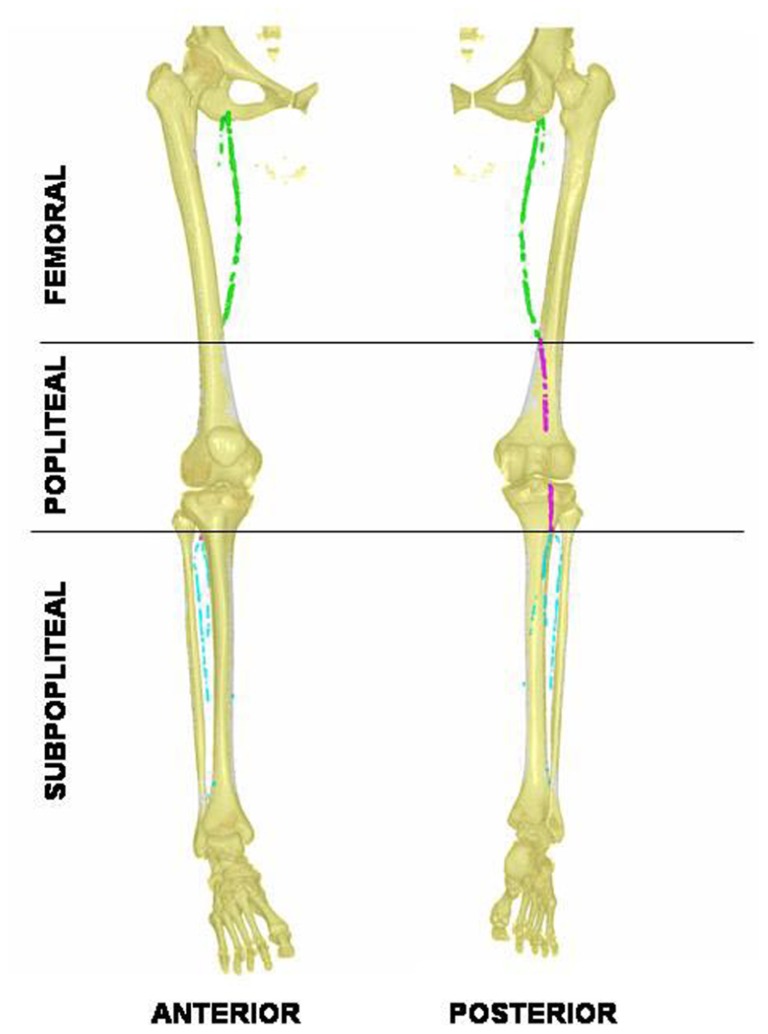
**Anterior and posterior 3D-views of lower limb arterial calcification in pseudoxanthoma elasticum revealed with helicoidal X-ray tomodensitometry**. Calcification in the femoral artery is tagged in green, the popliteal in purple, and the visible distal arteries are tagged in blue. Note the absence of calcifications within the middle popliteal segment.

The macroscopic distribution of the calcification along the arterial tree can be mapped using standard X-ray, although 3D reconstruction using helicoidal X-rays scans provides a more precise quantification and site identification (see **Figure 1**). In our experience, calcification accumulates mostly within the distal superficial femoral and below-knee arteries (tibialis and pedalis), a distribution mostly observed in the arteriosclerotic process associated with aging. Skin lesions are extensively documented in PXE, as skin biopsy are routinely done for diagnosis. However, histologic findings of the PXE-related vascular lesion are sparse and obtained from rare available autopsy samples. Ultrastructural analysis from the ascending aorta, iliac arteries, and vena cava from 2 males with PXE (36 and 80 years old), revealed that veins and arteries were similarly damaged (Gheduzzi et al., 2003). The alterations were not distributed homogeneously along the vessels with spotty alterations of elastic fibers, and aggregates of thin strands of amorphous elastin. The von Kossa staining revealed calcium sediment within the medial layers of the arterial wall of medium (e.g., carotids)- or small (radial)-sized arteries. In carotids, calcification was found extracellularly around elastin fibers although slight increase in intracellular calcium is also observed. Elastic fibers appears fragmented and proteoglycans accumulated preferentially within the media rather than intima compared to controls (Kornet et al., 2004). Qualitative and quantitative alteration in proteoglycans metabolism have been reported with increased heparin sulfate and decreased chondroitin sulfate in patient’s urine (Maccari et al., 2003).

## FUNCTIONAL CHANGES IN PXE ARTERY

The functional impacts of the arterial lesions, represented by elasto-calcinosis and proteoglycans accumulation, have been examined in a limited number of studies. Most of our knowledge is derived from non-invasive structural and dynamic observations using ultrasound techniques in living subjects and have provided important data for the understanding of the arterial lesions in PXE and their clinical expression. Three functional studies involving a small number (≈25) of PXE patients have focused on the carotid artery, an easily accessible large-sized musculo-elastic artery (Boutouyrie et al., 2001; Germain et al., 2003; Kornet et al., 2004). One of these studies has reported changes in the radial artery, a medium-sized muscular artery and the aorta, the main large-sized elastic artery of the body (Germain et al., 2003).

### INCREASED INTIMA MEDIA THICKNESS

An increased carotid intima media thickness (IMT) has been reported in two human studies (Germain et al., 2003; Kornet et al., 2004) and another more recently involving mice (Kupetsky-Rincon et al., 2012). Compared to age and gender-matched patients, these changes were more marked in older patients than in younger ones under the age of 40. An increased carotid IMT is independently associated with a higher risk for CV events which could represent a relevant argument to explain an accelerated arteriosclerosis in PXE with higher than normal CV incidents (Eigenbrodt et al., 2008). This increase in IMT was not associated with an enlargement of the lumen size and was responsible for a 19% increase in the arterial wall mass. By contrast, the radial arteries exhibited a smaller lumen with a thicker IMT, suggesting an inward remodeling (Germain et al., 2003).

The larger IMT seems to result from the higher amount of proteoglycans without proliferative change in the vascular smooth muscle cells (VSMC). Proliferation of the VSMC toward the lumen leads to arterial stenosis and occlusion, and is observed in response to an abnormal mechano-transductive signaling in elastin deficiency diseases, such as Williams–Beuren syndrome (Li et al., 1998). But this may not be the sole mechanism since a higher IMT with a progressive loss of VSMC has also been reported in Progeria (Gerhard-Herman et al., 2011). More complex changes have also been reported with multilayered aspects in PXE coronary (Miwa et al., 2004) or mammary arteries (Sarraj et al., 1999).

Although these conclusions remain to be confirmed in larger cohorts, the arterial remodeling in PXE peripheral arteries exhibits differences from other arterial remodeling such as aging, hypertension, and atherosclerosis. This remodeling is characterized by intima-media thickening predominantly in the large and medium-sized musculo-elastic arteries (Boutouyrie et al., 1992). Aging is also defined by an outward remodeling whereas hypertension and atherosclerosis are rather characterized by an inward remodeling. This remodeling is variable along the arterial tree (Bortolotto et al., 1999; Bjarnegard and Länne, 2010) and with gender, women exhibiting a higher wall/lumen ratio than men (Green et al., 2010). The use of imaging techniques with higher spatial resolution will be very helpful to confirm and discriminate the relative changes of thickness between the intimal and medial layers, and between the different vascular beds.

Arterial wall stiffness and elasticity in PXE arteries: It is expected that both fragmentation of elastic fibers and calcification will affect the arterial elasticity of the PXE arteries.

Compared to age and sex-matched controls, the distensibility was found either unchanged (Germain et al., 2003) or higher (Kornet et al., 2004) in the large-sized artery, such as carotid, and in medium-sized arteries such as radial (Germain et al., 2003). Furthermore, in the older female patients, the elastic modulus was found unchanged in small-sized but was higher in the medium-sized artery. A lower or unchanged compressibility of the small-sized arterial wall was also reported in the ankle arteries of PXE patients (Leftheriotis et al., 2011).

Therefore, it is likely that the complex rearrangement of the extracellular matrix in PXE arterial wall resulting from the combination of elastin fragmentation associated to proteoglycans replacement, focal accumulation of calcium and activated MMP (Zarbock et al., 2010) in the media could mask a predicted arterial stiffness.

## CLINICAL EXPRESSION OF THE VASCULAR PXE PHENOTYPE

The keystone clinical manifestations in PXE are represented by visual impairment due to the loss of central vision, large skin folds of esthetic concern, and CV complications (Neldner, 1988; Uitto et al., 2011). The vascular lesion in PXE takes a major place in the complications and the clinical outcome. Although, it seems that the lifespan of these patients is relatively preserved despite no clear reports on this point, the vascular impact of the disease is crucial and will be detailed in the following paragraphs.

### ARTERIAL HYPERTENSION

Since the earliest reports, the presence of an arterial hypertension in PXE has been controversial with a highly variable prevalence reported in the literature ranging from 8% (Neldner, 1988) and up to 25% of the patients (Gotting et al., 2005). Even for the highest range, this reported prevalence remains within the overall limits of hypertension estimated to be 26.4% of the world’s population (Kearney et al., 2005). In PXE, a higher than normal prevalence was likely explained on the basis of a renal artery stenosis or increased arterial stiffness resulting from the elasto-calcinosis (Goodman et al., 1963). A higher serum xylosyl-transferase (XT-1), a fibrosis marker of the extracellular matrix was found in hypertensive PXE patients (Gotting et al., 2005), though this is not supported by functional data (cf paragraph above). The association between ABCC6 and angiotensin polymorphisms (T174M and M235T) was not demonstrated in hypertensive PXE compared to normals (Gotting et al., 2005). Therefore, the higher prevalence of arterial hypertension in PXE remains to be demonstrated.

### ANEURYSMS AND DISSECTIONS

An abnormally dilated and/or ruptured arterial wall is the most life-threatening complication of the PXE disease. Contrary to other genetic diseases where the connective tissues are affected, as in Marfan’s or Elhers–Danlos diseases, reports of aneurysm in PXE patients are sparse and almost anecdotic.

In the cerebral vasculature, the prevalence of intracranial aneurysms is difficult to estimate. A Belgian PXE cohort (*n* = 100) based on self-reported data concluded that it was an unrelated association (van den Berg et al., 2000) while the association between intracranial malformations, including aneurysms, and PXE may not be fortuitous (Vasseur et al., 2011).

Aneurysms in the other vascular beds such as aorta or lower limbs are very rarely reported. ABCC6 mutations were found in a minority of non-PXE patients (5/133) with abdominal aortic aneurysms (Schulz et al., 2005), but this was not statistically significantly different from healthy controls and could not be considered as a genetic risk factor for aortic aneurysms. Aorto-coronary aneurysm has also been reported (Heno et al., 1998) but seems not specific to PXE as it has also been reported in other PXE-like syndromes, such as beta-thalassemia (Farmakis et al., 2004). Common genetic factors underlie medial calcification, such as ABCC6 and aneurysm development, suggesting that although medial disruption and calcification may occur in parallel, medial disruption does not strictly occur as a result of vascular calcification (Wang et al., 2009). The possibility for a higher prevalence for arterial dissection, such as the spontaneous disruption of the internal layers of an artery, common in the carotids of PXE, is still under discussion (Brandt et al., 2005), but remains anecdotic at present. Although several missense mutations (H623Q, R3190W, and R1268Q) were found in the patients with carotid dissection, these mutations were not disease-causing as they were also detected in healthy subjects (Morcher et al., 2003).

### ISCHEMIC STROKE

Beside the risk of stroke due to cerebral hemorrhage with ruptured intracranial aneurysms, the risk of ischemic stroke (IS) is another feared complication in PXE but remains difficult to establish. IS was reported in 15% of the PXE patients from a cohort of 38 patients compared to the general population (0.3–0.5%; Vanakker et al., 2008). In a cohort of 100 patients, IS was reported in seven patients with one patient having recurrent IS leading to a relative risk of 3.6 (95% confidence interval 3.3–4.0) of ISs in patients under 65 years (van den Berg et al., 2000). Focal cerebral ischemia in PXE was predominantly caused by small-vessel occlusive disease. Atherosclerotic plaques could co-exist with PXE lesions, but results from our cohort (unpublished data) showed that carotid plaques were absent in 55/93 (59.1%), unilateral in 17/93 (18.3%), and bilateral in 21/93 (22.6%) compared to age and gender-matched controls (*p* = 0.987) suggesting that it is not a primary mechanism for stroke in PXE. Transient cerebral ischemic attack could result from intermittent hemodynamic cerebral insufficiency due to intracranial arterial malformation (Vasseur et al., 2011).

### CARDIAC DISEASES

Cardiac diseases in PXE are mainly represented by myocardial infarction, angina pectoris, and valvular malfunction (Neldner, 1988; Vanakker et al., 2008). Data from the largest cohorts (Neldner, 1988; Vanakker et al., 2008) have reported symptoms of myocardial origin ranging from 13 to 15% for angina pectoris but lower for infarction (1–5%) of the patients occurring at age <55 years and sometimes causing death. In the coronary arterial bed, the association with a heterozygous R1141X mutation in ABCC6 and ischemic vascular events including stroke, was not demonstrated in the general population (*n* = 66831 participants; Hornstrup et al., 2011), although a strong association was reported only with coronary artery disease (Koblos et al., 2010). Additionally, Abcc6 deficiency was found to increase infarct size and apoptosis in a mouse cardiac ischemia–reperfusion model, although there were no differences in cardiac calcification following ischemia/reperfusion (Mungrue et al., 2012). Abnormal coronary wall suggests that specific structural factors are likely present in these vascular beds (Miwa et al., 2004). Interestingly, the transferability of the PXE phenotype, i.e., calcification, to the arterial graft is still questioned (Sarraj et al., 1999; Iliopoulos et al., 2002; Song et al., 2004).

### PERIPHERAL ARTERIAL DISEASE

Contrary to the other arterial beds, an early and severe peripheral arterial disease (PAD) described as a slowly worsening lower limb claudication is consistently and extensively reported in PXE patients without obvious CV risk factors. PAD is detected clinically by absence of ankle pulse, and the presence/absence of symptoms of a lower limb claudication such as a calf pain that limits or interrupts a walk. Additionally, the severity of PAD can be objectively determined by measuring the ankle-brachial systolic pressure index (ABI) which corresponds to the ratio of the systolic ankle and brachial pressures that normally ranges from 0.9 to 1.39, and the treadmill walking distance with the help of transcutaneous PO2 (Abraham et al., 2003). The prevalence of lower limb claudication, the symptomatic expression of PAD, is very high in PXE (53% in the Belgian cohort Vanakker et al., 2008 and 42% in ours) (Leftheriotis et al., 2011), a proportion clearly higher than the 9% men and 5% women with PAD reported in the general population. By contrast, the treadmill test, an objective evaluation of the arterial claudication, showed that only 56% of PXE with an ABI <0.90 were symptomatic during the test. The discrepancy between a high proportion of PAD detected by ABI with relatively less symptoms of intermittent claudication suggests that the PAD is well compensated by an efficient collateral circulation in PXE. This tolerance to ischemia was further demonstrated in our cohort by relatively well-preserved tissue oxygenation in PXE patients during walking. Interestingly, a relatively high incidence of subclinical peripheral artery disease (41%) was also reported in the carrier population (*n* = 21) suggesting that PAD could represent a frequent clinical manifestation, even in heterozygous patients. In addition to the presence/absence of ankle artery pulse, the ABI is a validated diagnostic tool for the detection of PAD and estimation of its severity, mainly in the asymptomatic patient (Diehm et al., 2009). PAD is also an independent marker of atherosclerosis associated with higher rates of CV diseases in the general population (Golomb et al., 2006). Calcifications in the tunica media of PXE patients are expected to increase arterial stiffness and thus decrease arterial wall compressibility (Kim et al., 2012). Although calcification predominates in the small-sized ankle arteries, the preserved arterial compressibility in these arteries remains unexplained.

### HEMORRHAGE

Hemorrhages are a frequent ophthalmologic complication in PXE due to the rupture of proliferative choroidal neovascularization secondary to angioid streak. To a lesser extent, gastrointestinal hemorrhage have also been reported and represent a life-threatening condition in PXE. The mechanism of hemorrhage remains unknown, but suggests sub-mucosal arterial malformations fragilized by the medial calcification with risk of rupture likely to the Bruch’s membrane in the eyes. Neovessels and/or malformation could also result from defective regulatory pathways and vascular endothelial growth factor (VEGF) gene polymorphisms such as the c.-460T and the c.674C alleles that are independent risk factors for development of severe retinopathy (Zarbock et al., 2009). The genetic deficiency gamma-glutamyl carboxylase (GGCX), which is a PXE-like syndrome associated with an abnormal production of coagulation factors, are more prone to severe uncontrol bleeding (Li et al., 2009a).

## WHAT DOES PXE MODELS TEACH US ABOUT CARDIOVASCULAR DISEASES?

Similar to other genetic diseases, the use of genetically engineered organisms such as mouse or zebrafish (*Danio rerio*) is helpful in our understanding of the PXE pathophysiology. Abcc6^-^^/^^-^ mice exhibit a low-to-mild level of medial arterial calcifications (Gorgels et al., 2005; Klement et al., 2005), although most calcification processes develop markedly in specific organs such as the vibrissae, and represents an interesting marker of peripheral calcification (Le Corre et al., 2012). Other mouse strains such as C3H/HeOuJ are spontaneously prone to soft connective tissues calcification, due to the defective function of Abcc6, that can be easily followed and quantified using X-rays (Le Corre et al., 2012). Finally, the zebrafish model exhibits severe abnormal development that were fully rescued by co-injection of mouse Abcc6 mRNA (Li et al., 2010). Although the mechanism of the arterial lesions in PXE remains unexplained, the fact that exteriorized tissue lesions develop remotely from the predominant sites of ABCC6 demonstrates that modifying factors are at play and limits our conclusions from these mouse models. There is now a constellation of data arguing for the concomitant role of modifying genes (Li et al., 2007; Hendig et al., 2008), polymorphisms (Schon et al., 2006) and mutations, regulatory pathways (Martin et al., 2011) as well as nutritional and environmental factors (Zarbock et al., 2007, 2009, 2010; Pisciotta et al., 2009) considerably widening the phenotypic expression and severity of the disease. Furthermore, the selective involvement of anatomical sites such as skin, eyes, arteries is usually explained by the presence of elastic fibers (with the exception of the lungs), but recent data suggests that collagen fibers could also be involved (Gorgels et al., 2012).

## THERAPEUTIC ISSUES IN THE PXE ARTERIAL DISEASE

In absence of validated and specific therapeutic targets in PXE, the treatment of arteriopathy remains limited. Vitamin K was not proven as an efficient treatment, although no data are presently available in human but the lack of a consistent result in proof-of-concept studies conducted in mice are not encouraging (Gorgels et al., 2011; Jiang et al., 2012). Despite disappointing initial results from a pilot study conducted in humans (Yoo et al., 2012), attempt to reduce calcification using phosphate binder (LaRusso et al., 2009) or magnesium supplementation has recently gained renewed interest with conclusive results in Abcc6^-^^/^^-^ mice (LaRusso et al., 2009; Li et al., 2009b; Kupetsky-Rincon et al., 2012). Treatment of acute ischemic complications is sporadically reported in the literature without obvious difference compared to the general population, although reports of limb amputation in the literature have never been made to our knowledge.

Finally, the careful management of CV risk factors remains an important point since atheroma could mask pre-existing lesions. Factors that could aggravate arterial calcification should be avoided in these patients such as anti-vitamin K which is an oral anticoagulant drug known to favor calcification (Price et al., 1998) that can now be advantageously replaced by anti-Xa oral therapy after the hemorrhage risk/benefit balance has been properly evaluated. The question of preventive anti-platelet therapy for IS is unsolved at present. In the general population, anti-platelet treatment such as aspirin is advised for secondary prevention of IS, but the higher prevalence of upper gastrointestinal hemorrhages in PXE remains a firm contraindication for the use of aspirin as well as for anticoagulants. A major challenge for all clinical trials attempted in PXE is the need for an objective and reproducible quantification of the soft-tissue calcification. In this way, quantification of the arterial calcification could be an interesting tool complementary to skin biopsy or ultrasound imaging and ophthalmologic follow-up since the severity of cutaneous manifestations and angioid streak of PXE are likely predictive of CV involvement (Utani et al., 2010).

In conclusion, although the arterial remodeling observed in PXE shares some of the features of arteriosclerosis and other calcifying vascular diseases of metabolic origin, it is not readily comparable to atherosclerosis. This raises challenging questions on the central role of the hepato-renal axis in the soft-tissue calcifying processes and PXE represents a prototypical systemic metabolic disease of genetic origin. The severity of the disease is progressive and highly variable in which CV symptoms and PAD seems a constant finding in these patients. Furthermore, the higher than expected compressibility of the arterial wall may represent a useful marker as well as quantification of the arterial calcium load. Data from larger cohorts are awaited with more detailed phenotypic descriptions. From a clinical point of view, the absence of efficient therapy, the follow-up of these patients first requires a tight control and management of the usual CV risk factors in addition to the limitation of pro-calcifying factors. The place of other anti-calcifying drugs, such as denosumab remains unknown at present. Finally a comparative analysis of the overlapping phenotypes such as PXE, generalized arterial calcification of infancy (GACI), and other PXE-like diseases is likely to add valuable information on the elusive mechanism of calcifying genetic diseases.

## Conflict of Interest Statement

The authors declare that the research was conducted in the absence of any commercial or financial relationships that could be construed as a potential conflict of interest.

## References

[B1] Abraham P. Picquet J. Vielle B. Sigaudo-Roussel D. Paisant-Thouveny F. Enon B. (2003). Transcutaneous oxygen pressure measurements on the buttocks during exercise to detect proximal arterial ischemia: comparison with arteriography. *Circulation* 107 1896–1900 12668524 10.1161/01.CIR.0000060500.60646.E0

[B2] Aessopos A. Samarkos M. Voskaridou E. Papaioannou D. Tsironi M. Kavouklis E. (1998). Arterial calcifications in beta-thalassemia. *Angiology* 49 137–143 9482513 10.1177/000331979804900206

[B3] Allam A. H. Thompson R. C. Wann L. S. Miyamoto M. I. Nur el-Din A. e.-H., el-Maksoud G. A. (2011). Atherosclerosis in ancient Egyptian mummies: the Horus study. *JACC Cardiovasc. Imaging* 4 315–327 21466986 10.1016/j.jcmg.2011.02.002

[B4] Assimes T. L. Knowles J. W. Basu A. Iribarren C. Southwick A. Tang H. (2008). Susceptibility locus for clinical and subclinical coronary artery disease at chromosome 9p21 in the multi-ethnic ADVANCE study. *Hum. Mol. Genet.* 17 2320–2328 18443000 10.1093/hmg/ddn132 PMC2733811

[B5] Atkinson J. (2008). Age-related medial elastocalcinosis in arteries: mechanisms, animal models, and physiological consequences. *J. Appl. Physiol.* 105 1643–1651 18772323 10.1152/japplphysiol.90476.2008

[B6] Baccarani-Contri M. Vincenzi D. Cicchetti F. Mori G. Pasquali-Ronchetti I. (1994). Immunochemical identification of abnormal constituents in the dermis of pseudoxanthoma elasticum patients. *Eur. J. Histochem.* 38 111–123 7524808

[B7] Becker A. Leber A. W. Becker C. von Ziegler F. Tittus J. Schroeder I. (2008). Predictive value of coronary calcifications for future cardiac events in asymptomatic patients with diabetes mellitus: a prospective study in 716 patients over 8 years. *BMC Cardiovasc. Disord.* 8:27 10.1186/1471-2261-8-27 PMC2569906 18847481

[B8] Bjarnegard N Länne T. (2010). Arterial properties along the upper arm in humans: age-related effects and the consequence of anatomical location. *J. Appl. Physiol.* 108 34–38 19875717 10.1152/japplphysiol.00479.2009

[B9] Bortolotto L. A. Hanon O. Franconi G. Boutouyrie P. Legrain S. Girerd X. (1999). The aging process modifies the distensibility of elastic but not muscular arteries. *Hypertension* 34 889–892 10523379 10.1161/01.hyp.34.4.889

[B10] Boutouyrie P. Germain D. P. Tropeano A. I. Laloux B. Carenzi F. Zidi M. (2001). Compressibility of the carotid artery in patients with pseudoxanthoma elasticum. *Hypertension* 38 1181–1184 11711519 10.1161/hy1101.096108

[B11] Boutouyrie P. Laurent S. Benetos A. Girerd X. J. Hoeks A. P. Safar M. E. (1992). Opposing effects of ageing on distal and proximal large arteries in hypertensives. *J. Hypertens. Suppl.* 10 S87-S91 1432336

[B12] Brampton C. Yamaguchi Y. Vanakker O. Van Laer L. Chen L. H. Thakore M. (2011). Vitamin K does not prevent soft tissue mineralization in a mouse model of pseudoxanthoma elasticum. *Cell Cycle* 10 1810–1820 21597330 10.4161/cc.10.11.15681 PMC3142464

[B13] Brandt T. Morcher M. Hausser I. (2005). Association of cervical artery dissection with connective tissue abnormalities in skin and arteries. *Front. Neurol. Neurosci.* 20 16–29 17290108 10.1159/000088131

[B14] Demer L. L. Tintut Y. (2008). Vascular calcification: pathobiology of a multifaceted disease. *Circulation* 117 2938–2948 18519861 10.1161/CIRCULATIONAHA.107.743161 PMC4431628

[B15] Diehm C. Allenberg J. R. Pittrow D. Mahn M. Tepohl G. Haberl R. L. (2009). Mortality and vascular morbidity in older adults with asymptomatic versus symptomatic peripheral artery disease. *Circulation* 120 2053–2061 19901192 10.1161/CIRCULATIONAHA.109.865600

[B16] Diekmann U. Zarbock R. Hendig D. Szliska C. Kleesiek K. Gotting C. (2009). Elevated circulating levels of matrix metalloproteinases MMP-2 and MMP-9 in pseudoxanthoma elasticum patients. *J. Mol. Med. (Berl.)*, 87 965–970 19575173 10.1007/s00109-009-0497-5

[B17] Eigenbrodt M. L. Bursac Z. Tracy R. E. Mehta J. L. Rose K. M. Couper D. J. (2008). B-mode ultrasound common carotid artery intima-media thickness and external diameter: cross-sectional and longitudinal associations with carotid atherosclerosis in a large population sample. *Cardiovasc. Ultrasound* 6 10 10.1186/1476-7120-6-10 PMC2277382 18321381

[B18] Fabbri E. Forni G. L. Guerrini G. Borgna-Pignatti C. (2009). Pseudoxanthoma-elasticum-like syndrome and thalassemia: an update. *Dermatol. Online J.* 15 7 19903435

[B19] Farmakis D. Vesleme V. Papadogianni A. Tsaftaridis P. Kapralos P. Aessopos A. (2004). Aneurysmatic dilatation of ascending aorta in a patient with beta-thalassemia and a pseudoxanthoma elasticum-like syndrome. *Ann. Hematol.* 83 596–599 15014899 10.1007/s00277-004-0859-6

[B20] Fülöp K. Jiang Q. Wetering K. V. D. Pomozi V. Szabo P. Aranyi T. (2011). ABCC6 does not transport vitamin K3-glutathione conjugate from the liver: relevance to pathomechanisms of pseudoxanthoma elasticum. *Biochem. Biophys. Res. Commun*. 415 468–471 22056557 10.1016/j.bbrc.2011.10.095 PMC3227420

[B21] Gerhard-Herman M. Smoot L. B. Wake N. Kieran M. W. Kleinman M. E. Miller D. T. (2011). Mechanisms of premature vascular aging in children with Hutchinson-Gilford Progeria Syndrome. *Hypertension* 59 92–97 22083160 10.1161/HYPERTENSIONAHA.111.180919 PMC3248242

[B22] Germain D. P. Boutouyrie P. Laloux B. Laurent S. (2003). Arterial remodeling and stiffness in patients with pseudoxanthoma elasticum. *Arterioscler. Thromb. Vasc. Biol.* 23 836–841 12649085 10.1161/01.ATV.0000067428.19031.28

[B23] Gheduzzi D. Sammarco R. Quaglino D. Bercovitch L. Terry S. Taylor W. (2003). Extracutaneous ultrastructural alterations in pseudoxanthoma elasticum. *Ultrastruct. Pathol.* 27 375–384 14660276

[B24] Giachelli C. M. (2004). Vascular calcification mechanisms. *J. Am. Soc. Nephrol.* 15 2959–2964 15579497 10.1097/01.ASN.0000145894.57533.C4

[B25] Golomb B. A. Dang T. T. Criqui M. H. (2006). Peripheral arterial disease: morbidity and mortality implications. *Circulation* 114 688–699 16908785 10.1161/CIRCULATIONAHA.105.593442

[B26] Goodman R. M. Smith E. W. Paton D. Bergman R. A. Siegel C. L. Ottesen O. E. (1963). Pseudoxanthoma elasticum: a clinical and histopathological study. *Medicine (Baltimore)* 42 297–334 14068068

[B27] Goodman W. G. Goldin J. Kuizon B. D. Yoon C. Gales B. Sider D. (2000). Coronary-artery calcification in young adults with end-stage renal disease who are undergoing dialysis. *N. Engl. J. Med.* 342 1478–1483 10816185 10.1056/NEJM200005183422003

[B28] Gorgels T. G. Hu X. Scheffer G. L. van der Wal A. C. Toonstra J. de Jong P. T. (2005). Disruption of Abcc6 in the mouse: novel insight in the pathogenesis of pseudoxanthoma elasticum. *Hum. Mol. Genet.* 14 1763–1773 15888484 10.1093/hmg/ddi183

[B29] Gorgels T. G. Teeling P. Meeldijk J. D. Nillesen S. T. van der Wal A. C. van Kuppevelt T. H. (2012). Abcc6 deficiency in the mouse leads to calcification of collagen fibers in Bruch’s membrane. *Exp. Eye Res.* 104 59–64 23041262 10.1016/j.exer.2012.09.009

[B30] Gorgels T. G. Waarsing J. H. Herfs M. Versteeg D. Schoensiegel F. Sato T. (2011). Vitamin K supplementation increases vitamin K tissue levels but fails to counteract ectopic calcification in a mouse model for pseudoxanthoma elasticum. *J. Mol. Med. (Berl.)* 89 1125–1135 21725681 10.1007/s00109-011-0782-y PMC3195265

[B31] Gotting C. Adam A. Szliska C. Kleesiek K. (2008). Circulating P-, L- and E-selectins in pseudoxanthoma elasticum patients. *Clin. Biochem.* 41 368–374 18191640 10.1016/j.clinbiochem.2007.12.009

[B32] Gotting C. Hendig D. Adam A. Schon S. Schulz V. Szliska C. (2005). Elevated xylosyltransferase I activities in pseudoxanthoma elasticum (PXE) patients as a marker of stimulated proteoglycan biosynthesis. *J. Mol. Med. (Berl.)* 83 984–992 16133423 10.1007/s00109-005-0693-x

[B33] Green D. J. Swart A. Exterkate A. Naylor L. H. Black M. A. Cable N. T. (2010). Impact of age, sex and exercise on brachial and popliteal artery remodelling in humans. *Atherosclerosis* 210 525–530 20197189 10.1016/j.atherosclerosis.2010.01.048

[B34] Hendig D. Langmann T. Kocken S. Zarbock R. Szliska C. Schmitz G. (2008). Gene expression profiling of ABC transporters in dermal fibroblasts of pseudoxanthoma elasticum patients identifies new candidates involved in PXE pathogenesis. *Lab. Invest.* 88 1303–1315 18936737 10.1038/labinvest.2008.96

[B35] Heno P. Fourcade L. Duc H. N. Bonello R. Roux O. Van de Walle J. P. (1998). [Aorto-coronary dysplasia and pseudoxanthoma elastica]. *Arch. Mal. Coeur Vaiss* 91 415–418 9749228

[B36] Hornstrup L. S. Tybjærg-Hansen A. Haase C. L. Nordestgaard B. R. G. Sillesen H. Grande P. (2011). Heterozygosity for R1141X in ABCC6 and risk of ischemic vascular disease/clinical perspective. *Circ. Cardiovasc. Genet.* 534–541 21831958 10.1161/CIRCGENETICS.110.958801

[B37] Hu X. Plomp A. S. van Soest S. Wijnholds J. de Jong P. T. Bergen A. A. (2003). Pseudoxanthoma elasticum: a clinical, histopathological, and molecular update. *Surv. Ophthalmol.* 48 424–438 12850230 10.1016/s0039-6257(03)00053-5

[B38] Iliopoulos J. Manganas C. Jepson N. Newman D. C. (2002). Pseudoxanthoma elasticum: is the left internal mammary artery a suitable conduit for coronary artery bypass grafting? *Ann. Thorac. Surg.* 73 652–653 11845895 10.1016/s0003-4975(01)03011-9

[B39] Jiang Q. Uitto J. (2006). Pseudoxanthoma elasticum: a metabolic disease? *J. Invest. Dermatol.* 126 1440–1441 16778810 10.1038/sj.jid.5700267

[B40] Jiang Q. Li Q. Grand-Pierre A. E. Schurgers L. J. Uitto J. (2012). Administration of vitamin K does not counteract the ectopic mineralization of connective tissues in Abcc6 (-/-) mice, a model for pseudoxanthoma elasticum. *Cell Cycle* 10 701–707 21304270 10.4161/cc.10.4.14862 PMC3173996

[B41] Jiang Q. Li Q. Uitto J. (2007). Aberrant mineralization of connective tissues in a mouse model of pseudoxanthoma elasticum: systemic and local regulatory factors. *J. Invest. Dermatol.* 127 1392–1402 17273159 10.1038/sj.jid.5700729

[B42] Jiang Q. Oldenburg R. Otsuru S. Grand-Pierre A. E. Horwitz E. M. Uitto J. (2010). Parabiotic heterogenetic pairing of Abcc6-/-/Rag1-/- mice and their wild-type counterparts halts ectopic mineralization in a murine model of pseudoxanthoma elasticum. *Am. J. Pathol.* 176 1855–1862 20185580 10.2353/ajpath.2010.090983 PMC2843475

[B43] Kalal I. G. Seetha D. Panda A. Nitschke Y. Rutsch F. (2012). Molecular diagnosis of generalized arterial calcification of infancy (GACI). *J. Cardiovasc. Dis. Res.* 3 150–154 22629037 10.4103/0975-3583.95373 PMC3354462

[B44] Kearney P. M. Whelton M. Reynolds K. Muntner P. Whelton P. K. He J. (2005). Global burden of hypertension: analysis of worldwide data. *Lancet* 365 217–223 15652604 10.1016/S0140-6736(05)17741-1

[B45] Kim H. G. Park S. C. Lee S. L. Shin O. R. Yoon S. A. Yang C. W. (2012). Arterial micro-calcification of vascular access is associated with aortic arch calcification and arterial stiffness in hemodialysis patients. *Semin. Dial.* 10.1111/j.1525-139X.2012.01113.x [Epub ahead of print] 22909025

[B46] Klement J. F. Matsuzaki Y. Jiang Q. J. Terlizzi J. Choi H. Y. Fujimoto N. (2005). Targeted ablation of the abcc6 gene results in ectopic mineralization of connective tissues. *Mol. Cell. Biol.* 25 8299–8310 16135817 10.1128/MCB.25.18.8299-8310.2005 PMC1234326

[B47] Koblos G. Andrikovics H. Prohaszka Z. Tordai A. Varadi A. Aranyi T. (2010). The R1141X loss-of-function mutation of the ABCC6 gene is a strong genetic risk factor for coronary artery disease. *Genet. Test. Mol. Biomarkers* 14 75–78 19929409 10.1089/gtmb.2009.0094 PMC2935842

[B48] Kool M. van der Linden M. de Haas M. Baas F. Borst P. (1999). Expression of human MRP6, a homologue of the multidrug resistance protein gene MRP1, in tissues and cancer cells. *Cancer Res.* 59 175–182 9892204

[B49] Kornet L. Bergen A. A. Hoeks A. P. Cleutjens J. P. Oostra R. J. Daemen M. J. (2004). In patients with pseudoxanthoma elasticum a thicker and more elastic carotid artery is associated with elastin fragmentation and proteoglycans accumulation. *Ultrasound Med. Biol.* 30 1041–1048 15474747 10.1016/j.ultrasmedbio.2004.06.004

[B50] Kupetsky-Rincon E. A. Li Q. Uitto J. (2012). Magnesium reduces carotid intima-media thickness in a mouse model of pseudoxanthoma elasticum: a novel treatment biomarker. *Clin. Transl. Sci.* 5 259–264 22686203 10.1111/j.1752-8062.2011.00390.x PMC3572782

[B51] LaRusso J. Li Q. Jiang Q. Uitto J. (2009). Elevated dietary magnesium prevents connective tissue mineralization in a mouse model of pseudoxanthoma elasticum (Abcc6(-/-)). *J. Invest. Dermatol.* 129 1388–1394 19122649 10.1038/jid.2008.391 PMC2879622

[B52] Laurent S. Briet M. Boutouyrie P. (2009). Large and small artery cross-talk and recent morbidity-mortality trials in hypertension. *Hypertension* 54 388–392 19546376 10.1161/HYPERTENSIONAHA.109.133116

[B53] Le Corre Y. Le Saux O. Froeliger F. Libouban H. Kauffenstein G. Willoteaux S. (2012). Quantification of the calcification phenotype of Abcc6-deficient mice with microcomputed tomography. *Am. J. Pathol.* 180 2208–2213 22469843 10.1016/j.ajpath.2012.02.007 PMC5691325

[B54] Leftheriotis G. Abraham P. Le Corre Y. Le Saux O. Henrion D. Ducluzeau P. H. (2011). Relationship between ankle brachial index and arterial remodeling in pseudoxanthoma elasticum. *J. Vasc. Surg.* 54 1390–1394 21723076 10.1016/j.jvs.2011.04.041 PMC5529101

[B55] Le Saux O. Bunda S. VanWart C. M. Douet V. Got L. Martin L. (2006). Serum factors from pseudoxanthoma elasticum patients alter elastic fiber formation in vitro. *J. Invest. Dermatol.* 126 1497–1505 16543900 10.1038/sj.jid.5700201 PMC5540375

[B56] Le Saux O. Urban Z. Tschuch C. Csiszar K. Bacchelli B. Quaglino D. (2000). Mutations in a gene encoding an ABC transporter cause pseudoxanthoma elasticum. *Nat. Genet.* 25 223–227 10835642 10.1038/76102

[B57] Li D. Y. Brooke B. Davis E. C. Mecham R. P. Sorensen L. K. Boak B. B. (1998). Elastin is an essential determinant of arterial morphogenesis. *Nature* 393 276–280 9607766 10.1038/30522

[B58] Li Q. Grange D. K. Armstrong N. L. Whelan A. J. Hurley M. Y. Rishavy M. A. (2009a). Mutations in the GGCX and ABCC6 genes in a family with pseudoxanthoma elasticum-like phenotypes. *J. Invest. Dermatol.* 129 553–563 18800149 10.1038/jid.2008.271 PMC2900916

[B59] Li Q. Larusso J. Grand-Pierre A. E. Uitto J. (2009b). Magnesium carbonate-containing phosphate binder prevents connective tissue mineralization in Abcc6(-/-) mice-potential for treatment of pseudoxanthoma elasticum. *Clin. Transl. Sci.* 2 398–404 20443931 10.1111/j.1752-8062.2009.00161.x PMC3005270

[B60] Li Q. Guo H. Chou D. Harrington D. Schurgers L. J. Terry S. F. (2012). “Warfarin accelerates the ectopic mineralization in Abcc6-/- mice – clinical relevance to pseudoxanthoma elsaticum,” in *2012 PXE Research Meeting*, September 24, Bethesda 10.1016/j.ajpath.2012.12.037 PMC3620423 23415960

[B61] Li Q. Jiang Q. Larusso J. Klement J. F. Sartorelli A. C. Belinsky M. G. (2007). Targeted ablation of Abcc1 or Abcc3 in Abcc6(-/-) mice does not modify the ectopic mineralization process. *Exp. Dermatol.* 16 853–859 17845218 10.1111/j.1600-0625.2007.00621.x

[B62] Li Q. Sadowski S. Frank M. Chai C. Varadi A. Ho S. Y. (2010). The abcc6a gene expression is required for normal zebrafish development. *J. Invest. Dermatol.* 130 2561–2568 20596085 10.1038/jid.2010.174 PMC3357064

[B63] Maccari F. Gheduzzi D. Volpi N. (2003). Anomalous structure of urinary glycosaminoglycans in patients with pseudoxanthoma elasticum. *Clin. Chem.* 49 380–388 12600949 10.1373/49.3.380

[B64] Mackenzie N. C. Huesa C. Rutsch F. Macrae V. E. (2012). New insights into NPP1 function: lessons from clinical and animal studies. *Bone* 51 961–968 22842219 10.1016/j.bone.2012.07.014

[B65] Markello T. C. Pak L. K. St Hilaire C. Dorward H. Ziegler S. G. Chen M. Y. (2011). Vascular pathology of medial arterial calcifications in NT5E deficiency: implications for the role of adenosine in pseudoxanthoma elasticum. *Mol. Genet. Metab.* 103 44–50 21371928 10.1016/j.ymgme.2011.01.018 PMC3081917

[B66] Martin L. Douet V. VanWart C. M. Heller M. B Le Saux O. (2011). A mouse model of beta-thalassemia shows a liver-specific down-regulation of Abcc6 expression. *Am. J. Pathol.* 178 774–783 21281810 10.1016/j.ajpath.2010.10.004 PMC3069908

[B67] Martin L. J. Lau E. Singh H. Vergnes L. Tarling E. J. Mehrabian M. (2012). ABCC6 localizes to the mitochondria-associated membrane. *Circ. Res.* 111 516–520 22811557 10.1161/CIRCRESAHA.112.276667 PMC3540978

[B68] Miwa K. Higashikata T. Mabuchi H. (2004). Intravascular ultrasound findings of coronary wall morphology in a patient with pseudoxanthoma elasticum. *Heart* 90 e61 10.1136/hrt.2004.040592 PMC1768477 15367534

[B69] Morcher M. Hausser I. Brandt T. Grond-Ginsbach C. (2003). Heterozygous carriers of Pseudoxanthoma elasticum were not found among patients with cervical artery dissections. *J. Neurol.* 250 983–986 12928920 10.1007/s00415-003-1139-4

[B70] Mungrue I. N. Zhao P. Yao Y. Meng H. Rau C. Havel J. V. (2012). Abcc6 deficiency causes increased infarct size and apoptosis in a mouse cardiac ischemia-reperfusion model. *Arterioscler. Thromb. Vasc. Biol.* 31 2806–2812 21979437 10.1161/ATVBAHA.111.237420 PMC3227394

[B71] Musallam K. M. Beydoun A. Hourani R. Nasreddine W. Raad R. Koussa S. (2011). Brain magnetic resonance angiography in splenectomized adults with beta-thalassemia intermedia. *Eur. J. Haematol.* 87 539–546 21913989 10.1111/j.1600-0609.2011.01706.x

[B72] Neldner K. H. (1988). Pseudoxanthoma elasticum. *Clin. Dermatol.* 6 1–159 3359381 10.1016/0738-081x(88)90003-x

[B73] Nitschke Y. Baujat G. Botschen U. Wittkampf T. du Moulin M. Stella J. (2012). Generalized arterial calcification of infancy and pseudoxanthoma elasticum can be caused by mutations in either ENPP1 or ABCC6. *Am. J. Hum. Genet.* 90 25–39 22209248 10.1016/j.ajhg.2011.11.020 PMC3257960

[B74] O’Donnell C. J. Chazaro I. Wilson P. W. Fox C. Hannan M. T. Kiel D. P. (2002). Evidence for heritability of abdominal aortic calcific deposits in the Framingham Heart Study. *Circulation* 106 337–341 12119250 10.1161/01.cir.0000022663.26468.5b

[B75] O’Rourke M. F. Staessen J. A. Vlachopoulos C. Duprez D. Plante G. E. (2002). Clinical applications of arterial stiffness; definitions and reference values. *Am. J. Hypertens.* 15 426–444 12022246 10.1016/s0895-7061(01)02319-6

[B76] Pasquali-Ronchetti I. Garcia-Fernandez M. I. Boraldi F. Quaglino D. Gheduzzi D. De Vincenzi Paolinelli C. (2006). Oxidative stress in fibroblasts from patients with pseudoxanthoma elasticum: possible role in the pathogenesis of clinical manifestations. *J. Pathol.* 208 54–61 16261549 10.1002/path.1867

[B77] Persy V D’Haese P. (2009). Vascular calcification and bone disease: the calcification paradox. *Trends Mol. Med.* 15 405–416 19733120 10.1016/j.molmed.2009.07.001

[B78] Pisciotta L. Tarugi P. Borrini C. Bellocchio A. Fresa R. Guerra D. (2009). Pseudoxanthoma elasticum and familial hypercholesterolemia: a deleterious combination of cardiovascular risk factors. *Atherosclerosis* 210 173–176 20018285 10.1016/j.atherosclerosis.2009.11.028

[B79] Price P. A. Faus S. A. Williamson M. K. (1998). Warfarin causes rapid calcification of the elastic lamellae in rat arteries and heart valves. *Arterioscler. Thromb. Vasc. Biol.* 18 1400–1407 9743228 10.1161/01.atv.18.9.1400

[B80] Quaglino D. Boraldi F. Barbieri D. Croce A. Tiozzo R Pasquali Ronchetti I. (2000). Abnormal phenotype of in vitro dermal fibroblasts from patients with Pseudoxanthoma elasticum (PXE). *Biochim. Biophys. Acta* 1501 51–62 10727849 10.1016/s0925-4439(00)00007-7

[B81] Rampersaud E. Bielak L. F. Parsa A. Shen H. Post W. Ryan K. A. (2008). The association of coronary artery calcification and carotid artery intima-media thickness with distinct, traditional coronary artery disease risk factors in asymptomatic adults. *Am. J. Epidemiol.* 168 1016–1023 18805900 10.1093/aje/kwn211 PMC2720772

[B82] Ratnavel R. C. Norris P. G. (1994). Penicillamine-induced elastosis perforans serpiginosa treated successfully with isotretinoin. *Dermatology* 189 81–83 8003796 10.1159/000246792

[B83] Rutsch F. Nitschke Y. Terkeltaub R. (2011). Genetics in arterial calcification: pieces of a puzzle and cogs in a wheel. *Circ. Res.* 109 578–592 21852556 10.1161/CIRCRESAHA.111.247965 PMC3248761

[B84] Rutsch F. Vaingankar S. Johnson K. Goldfine I. Maddux B. Schauerte P. (2001). PC-1 nucleoside triphosphate pyrophosphohydrolase deficiency in idiopathic infantile arterial calcification. *Am. J. Pathol.* 158 543–554 11159191 10.1016/S0002-9440(10)63996-X PMC1850320

[B85] Sarraj A. Al Homsi M. F. Khouqeer F. (1999). Pseudoxanthoma elasticum of the internal mammary artery. *Cardiovasc. Surg.* 7 381–384 10386762 10.1177/096721099900700321

[B86] Schon S. Schulz V. Prante C. Hendig D. Szliska C. Kuhn J. (2006). Polymorphisms in the xylosyltransferase genes cause higher serum XT-I activity in patients with pseudoxanthoma elasticum (PXE) and are involved in a severe disease course. *J. Med. Genet.* 43 745–759 16571645 10.1136/jmg.2006.040972 PMC2593031

[B87] Schulz V. Hendig D. Schillinger M. Exner M. Domanovits H. Raith M. (2005). Analysis of sequence variations in the ABCC6 gene among patients with abdominal aortic aneurysm and pseudoxanthoma elasticum. *J. Vasc. Res.* 42 424–432 16127278 10.1159/000087900

[B88] Shanahan C. M. Cary N. R. B. Salisbury J. R. Proudfoot D. Weissberg P. L. Edmonds M. E. (1999). Medial localization of mineralization-regulating proteins in association with Monckeberg’s sclerosis: evidence for smooth muscle cell-mediated vascular calcification. *Circulation* 100 2168–2176 10571976 10.1161/01.cir.100.21.2168

[B89] Simionescu A. Philips K. Vyavahare N. (2005). Elastin-derived peptides and TGF-beta1 induce osteogenic responses in smooth muscle cells. *Biochem. Biophys. Res. Commun.* 334 524–532 16005428 10.1016/j.bbrc.2005.06.119

[B90] Song H. K. Sharoni E. Williams B. Jr. Guyton R. A. Puskas J. D. (2004). Long-term left internal mammary artery graft patency for coronary artery disease associated with pseudoxanthoma elasticum. *Ann. Thorac. Surg.* 78 691–693 15276551 10.1016/j.athoracsur.2003.08.018

[B91] Hilaire St. C. Ziegler S. G. Markello T. C. Brusco A. Groden C. Gill F. (2011). NT5E mutations and arterial calcifications. *N. Engl. J. Med.* 364 432–442 21288095 10.1056/NEJMoa0912923 PMC3049958

[B92] Uitto J. Bercovitch L. Terry S. F. Terry P. F. (2011). Pseudoxanthoma elasticum: progress in diagnostics and research towards treatment: summary of the 2010 PXE International Research Meeting. *Am. J. Med. Genet. A* 155A 1517–1526 21671388 10.1002/ajmg.a.34067 PMC3121926

[B93] Uitto J. Li Q. Jiang Q. (2010). Pseudoxanthoma elasticum: molecular genetics and putative pathomechanisms. *J. Invest. Dermatol.* 130 661–670 20032990 10.1038/jid.2009.411 PMC3349438

[B94] Utani A. Tanioka M. Yamamoto Y. Taki R. Araki E. Tamura H. (2010). Relationship between the distribution of pseudoxanthoma elasticum skin and mucous membrane lesions and cardiovascular involvement. *J. Dermatol.* 37 130–136 20175846 10.1111/j.1346-8138.2009.00775.x

[B95] Vanakker O. M. Leroy B. P. Coucke P. Bercovitch L. G. Uitto J. Viljoen D. (2008). Novel clinico-molecular insights in pseudoxanthoma elasticum provide an efficient molecular screening method and a comprehensive diagnostic flowchart. *Hum. Mutat.* 29 205 10.1002/humu.9514 18157818

[B96] Vanakker O. M. Leroy B. P. Schurgers L. J. Vermeer C. Coucke P. J De Paepe A. (2011). Atypical presentation of pseudoxanthoma elasticum with abdominal cutis laxa: evidence for a spectrum of ectopic calcification disorders? *Am. J. Med. Genet. A* 155A 2855–2859 21964806 10.1002/ajmg.a.34264

[B97] Vanakker O. M. Martin L. Gheduzzi D. Leroy B. P. Loeys B. L. Guerci V. I. (2007). Pseudoxanthoma elasticum-like phenotype with cutis laxa and multiple coagulation factor deficiency represents a separate genetic entity. *J. Invest. Dermatol.* 127 581–587 17110937 10.1038/sj.jid.5700610

[B98] van den Berg J. S. Hennekam R. C. Cruysberg J. R. Steijlen P. M. Swart J. Tijmes N. (2000). Prevalence of symptomatic intracranial aneurysm and ischaemic stroke in pseudoxanthoma elasticum. *Cerebrovasc. Dis.* 10 315–319 10878438 10.1159/000016076

[B99] Vasseur M. Carsin-Nicol B. Ebran J. M. Willoteaux S. Martin L. Leftheriotis G. (2011). Carotid rete mirabile and pseudoxanthoma elasticum: an accidental association? *Eur. J. Vasc. Endovasc. Surg.* 42 292–294 21723754 10.1016/j.ejvs.2011.05.007

[B100] Wang C. Li Y. Shi L. Ren J. Patti M. Wang T. (2012). Mutations in SLC20A2 link familial idiopathic basal ganglia calcification with phosphate homeostasis. *Nat. Genet.* 44 254–256 22327515 10.1038/ng.1077

[B101] Wang S. S. Martin L. J. Schadt E. E. Meng H. Wang X. Zhao W. (2009). Disruption of the aortic elastic lamina and medial calcification share genetic determinants in mice. *Circ. Cardiovasc. Genet.* 2 573–582 20031637 10.1161/CIRCGENETICS.109.860270 PMC2836127

[B102] Yoo J. Y. Blum R. R. Singer G. K. Stern D. K. Emanuel P. O. Fuchs W. (2012). A randomized controlled trial of oral phosphate binders in the treatment of pseudoxanthoma elasticum. *J. Am. Acad. Dermatol.* 65 341–348 21496949 10.1016/j.jaad.2010.05.023

[B103] Zarbock R. Hendig D. Szliska C. Kleesiek K. Gotting C. (2007). Pseudoxanthoma elasticum: genetic variations in antioxidant genes are risk factors for early disease onset. *Clin. Chem.* 53 1734–1740 17693525 10.1373/clinchem.2007.088211

[B104] Zarbock R. Hendig D. Szliska C. Kleesiek K. Gotting C. (2009). Vascular endothelial growth factor gene polymorphisms as prognostic markers for ocular manifestations in pseudoxanthoma elasticum. *Hum. Mol. Genet.* 18 3344–3351 19483196 10.1093/hmg/ddp259

[B105] Zarbock R. Hendig D. Szliska C. Kleesiek K Götting C. (2010). Analysis of MMP2 promoter polymorphisms in patients with pseudoxanthoma elasticum. *Clin. Chim. Acta* 411 1487–1490 20541540 10.1016/j.cca.2010.06.006

